# CoQ_10_ reduces glioblastoma growth and infiltration through proteome remodeling and inhibition of angiogenesis and inflammation

**DOI:** 10.1007/s13402-022-00734-0

**Published:** 2022-11-02

**Authors:** Javier Frontiñán-Rubio, Emilio Llanos-González, Sonia García-Carpintero, Juan Ramón Peinado, Inmaculada Ballesteros-Yáñez, Margarita Villar Rayo, José de la Fuente, Víctor M. Pérez-García, Luis A. Perez-Romasanta, Marcos Malumbres, Francisco J. Alcaín, Mario Durán-Prado

**Affiliations:** 1 Department of Medical Sciences, Faculty of Medicine, University of Castilla-La Mancha, 13071 Ciudad Real, Spain; 2 Oxidative Stress and Neurodegeneration Group, Faculty of Medicine, Regional Centre for Biomedical Research, University of Castilla-La Mancha, Ciudad Real, Spain; 3 EMAS Group, Faculty of Medicine, Regional Centre for Biomedical Research, University of Castilla-La Mancha, Ciudad Real, Spain; 4 SaBio Research Group, Hunting Resources Research Institute (IREC), Ciudad Real, Spain; 5 Laboratory of Mathematical Oncology, University of Castilla-La Mancha, Ciudad Real, Spain; 6 Radiology and Medicinal Physics, Institute for Biomedical Research of Salamanca (IBSAL), Salamanca, Spain; 7 Cell Division and Cancer Group, Spanish National Cancer Research Centre (CNIO), Madrid, Spain

**Keywords:** Angiogenesis, Coenzyme Q_10_, Glioblastoma, Inflammation, Invasion

## Abstract

**Purpose:**

Most monotherapies available against glioblastoma multiforme (GBM) target individual hallmarks of this aggressive brain tumor with minimal success. In this article, we propose a therapeutic strategy using coenzyme Q_10_ (CoQ_10_) as a pleiotropic factor that crosses the blood–brain barrier and accumulates in cell membranes acting as an antioxidant, and in mitochondrial membranes as a regulator of cell bioenergetics and gene expression.

**Methods:**

Xenografts of U251 cells in nu/nu mice were used to assay tumor growth, hypoxia, angiogenesis, and inflammation. An orthotopic model was used to explore microglial infiltration, tumor growth, and invasion into the brain parenchyma. Cell proliferation, migration, invasion, proteome remodeling, and secretome were assayed in vitro. Conditioned media were used to assay angiogenesis, monocyte chemoattraction, and differentiation into macrophages in vitro.

**Results:**

CoQ_10_ treatment decreased tumor volume in xenografts and orthotopic models, although its effect on tumor cell proliferation was not direct. Tumors from mice treated with CoQ_10_ were less hypoxic and vascularized, having less infiltration from inflammatory cells. Treatment-induced downregulation of HIF-1α and NF-kB led to a complete remodeling of the tumor cells proteome and secretome, impacting angiogenesis, monocyte infiltration, and their differentiation into macrophages. Besides, tumor cell migration and invasion were drastically restricted by mechanisms involving modulation of the actin cytoskeleton and downregulation of matrix metalloproteases (MMPs).

**Conclusions:**

CoQ_10_ has a pleiotropic effect on GBM growth, targeting several hallmarks simultaneously. Thus, its integration into current treatments of this fatal disease should be considered.

**Supplementary Information:**

The online version contains supplementary material available at 10.1007/s13402-022-00734-0.

## Introduction

Glioblastoma (GBM), the most common type of malignant brain tumor in adults, is associated with a median overall survival of 15 months when receiving the actual standard of care—the Stupp protocol, established in 2005 [1, 2]. Since that date, there have been minor advances in the treatment of this devasting pathology—i.e., bevacizumab, tumor treating fields, immunotherapy, and other mono-target therapies—which have helped increase median overall survival up to 18–20 months [2]. Current therapies fail partly due to tumor heterogeneity, low CNS drug penetration [3], and resistance to radiotherapy due to imbalanced redox homeostasis [4], motivating the search for novel strategies to overcome the particularities of these tumors.

GBM is characterized by an elevated proliferative rate and a high infiltrative capacity [2]. HIF-1α plays a key role in its aggressive characteristics [5], governing the transcription of hundreds of genes responsible for cell death avoidance and promoting genetic instability, neoangiogenesis, inflammation, immune response evasion, invasion, and changes in transcriptional signaling pathways such as PI3K/Akt and Nf-κB [5–7], accompanied by a switch from oxidative phosphorylation to glycolytic glucose metabolism and promotion of the pentose phosphate pathway [8]. Hypoxia appears during tumor growth, promoting neoangiogenesis and inflammation, which feed cell proliferation and invasion—thus creating a cyclic response [9].

We have previously shown that treatment of GBM cells with CoQ_10_ acts as a radiosensitizer, targeting the naturally increased tumor-associated mitochondrial reactive oxygen species (ROS) and diminishing the total antioxidant cell capacity by mechanisms involving a reduction in the level of HIF-1α [10]. CoQ_10_ is a well-known cellular antioxidant and a component of the mitochondrial electron transport chain that crosses the blood–brain barrier [11]. Indeed, its therapeutic potential is being exploited in the treatment of different pathologies such as neurodegenerative diseases or cancer [12–14]. CoQ_10_ exerts multiple pleiotropic biological activities apart from radical scavenging, such as immune-boosting [12].

In this work, we have explored the potential of CoQ10 in xenografts and orthotopic models of GBM, analyzing its effect at the molecular and cellular levels in vitro. Our results show how treatment with CoQ_10_ reduced tumor size in subcutaneous GBM xenoimplants and orthotopic models. However, the effect is neither due to direct inhibition of cell proliferation nor the induction of apoptosis, as in vitro experiments revealed a low impact on GBM cells. The action of CoQ_10_ is more complex and involves short-term effects—i.e., remodeling the cell proteome, which subsequently impacts the secretome. CoQ_10_ reduces HIF-1α, p-Akt, and NF-κB levels, regulating the processes of tumor neovascularization, inflammation, and tumor cell invasion. Our results suggest that CoQ10 could be potentially interesting for the clinical management of GBM, as it is cheap, can be orally administered, has no side effects, and can target several hallmarks of the pathology simultaneously.

## Material and methods

### Cell culture and CoQ_10_ treatment

Human GBM U251 and human monocytic leukemia Thp-1 cells were obtained from ATCC. Human umbilical vein endothelial cells (HUVEC) were obtained from Lonza (C2517A). Cells were cultured at 37℃ and 5% CO_2_. U251 and Thp-1 were maintained in DMEM (#D5796; Sigma-Aldrich) and RPMI-1640 (#R8758; Sigma-Aldrich) respectively, with 10% fetal bovine serum (FBS) (#F4135; Sigma-Aldrich) and 1% antibiotic/antimycotic (#A5955; Sigma-Aldrich). HUVECs were grown in EBM-2 Basal Medium (CC-3156) and with EGM-2 SingleQuots Supplements (CC-4176). CoQ_10_ was generously provided by Kaneka Corporation. When indicated, cells were treated for 24 h with 5 µM CoQ_10_ or vehicle (ethanol; control).

### Mice models

Xenograft and orthotopic implantation studies were performed with athymic nu/nu mice (Envigo). Mice were fed with autoclaved pelleted food and water ad libitum. Tumor generation, measurement, and processing were performed as previously described [15–17]. For xenograft, U251 cells mixed in Matrigel (1:1) (A1413201; Thermo Fisher) were injected subcutaneously into the flank of each mouse. Tumor volume was calculated every week using a digital caliper. After twelve weeks, animals were sacrificed, and the tumor samples were collected and processed (n = 7; 4 Vh & 3 CoQ_10_).

For orthotopic intracranial implantation, mice were anesthetized with an intraperitoneal injection of ketamine and xylazine. The animals were placed in a Kopf (Tujunga, CA) stereotaxic apparatus, and the skull trepanned at the injection spot into the striatum (0.5 mm in front of the Bregma, 2.1 mm lateral position, and 3 mm ventral position from the dura). U251 cells (3 × 10^5^ cells in a volume of 2 μL) were implanted intracranially using a micro syringe (10 μL NeurosModel 1701 RN, point style 4, SYR, Hamilton Co.). The animals were housed on a standard 12/12 h light/dark cycle at 21 °C with food and water ad libitum. Once the different models were established, the treatment with CoQ_10_ (100 mg/Kg mouse) or vehicle (saline solution) was performed intraperitoneal, every four days, starting on day seven. Tumor-bearing animals (n = 9; 4 Vh & 5 CoQ_10_) were sacrificed at week 9. Animal procedures followed European (Directive 2010/63/EU) and Spanish (RO 53/2013) legislation on the protection of animals used for scientific purposes. Experiments were previously approved by the Ethical Committee of Animal Research of the University of Castilla-La Mancha (PR-2012–6-03).

### Immunofluorescence and immunohistochemical procedures

Immunohistochemistry and immunofluorescence were performed as standard protocols previously described [14, 18]. Images were captured through an LSM 710 Zeiss confocal microscope (Oberkochen, Germany) and an Eclipse TiU inverted microscope (Nikon, Tokyo, Japan). All data were processed and analyzed using ImageJ 1.53 software (National Institutes of Health [NIH], Bethesda, USA). Supplementary Materials and Methods provide a complete description of antibodies and methodology.

### Hypoxia quantification

Hypoxia was measured using Hypoxyprobe (pimonidazole hydrochloride) as previously described [19]. Briefly, before the animals were sacrificed, they were injected with pimonidazole, a compound that forms adducts in hypoxic regions, which can then be examined by fluorescence microscopy by immunostaining. Once mice were sacrificed, tissues were processed, and the adducts generated by hypoxia were imaged by confocal microscopy or a Cytation 5 cell multimode reader and quantified with ImageJ.

### Tumor volume estimation

The area of the primary tumor and infiltration was determined through vimentin immunostaining. Volumetric values were obtained using serial cuts, applying the Cavalieri estimator method [20]. The same approach was used to calculate the maximum volume of infiltration. A full description of the methodology is provided in Supplementary Materials and Methods.

### Proteomics

Proteomic studies were performed as previously described [17]. The proteome was assessed in a RP-LC–MS/MS using an Easy-nLC II system coupled to a LCQ Fleet ion trap mass spectrometer (Thermo Scientific). Peptides were detected in survey scans from 400 to 1600 amu (1 μscan), followed by three data-dependent MS/MS scans (Top 3), using an isolation width of 2 mass-to-charge ratio units. Clustering and paired analysis for changes in protein levels were assessed with the free software MEV 4.9.

### Immunocytochemistry

Cells were treated for 24 h with 5 µM CoQ_10_ or vehicle (ethanol). Then cells were fixed for 15 min in 4% paraformaldehyde (PFA), blocked, and stained with different primary antibodies (full description in Supplementary Materials). Primary antibody binding was detected using secondary antibodies conjugated with Alexa 488 (goat anti-mouse A32723; goat anti-rabbit A32731; rabbit anti-goat A-11078, Life Technologies) and Alexa 594 (goat anti-mouse A11005; goat anti-rabbit A11012; Life Technologies). Images were captured through an Eclipse TiU inverted microscope (Nikon, Tokyo, Japan). All data were processed and analyzed using ImageJ 1.53 software (NIH, Bethesda, USA).

### Western blotting

Western blot was performed as previously described [10]. Briefly, cells were treated for 24 h with 5 µM CoQ_10_ or vehicle (ethanol). Then, cells were washed with PBS and lysed in RIPA buffer or SDS-DTT buffer. Lysates were obtained by centrifugation and quantified using BCA protein assay kit (71,285-M; Merck). Equal amounts of protein were separated using 10% or 12% acrylamide gel, and the proteins were transferred to a nitrocellulose membrane. Then, membranes were incubated with primary antibodies (see Supplementary Materials). After that, membranes were washed and incubated with secondary antibodies goat anti-rabbit (P0448; Dako), goat anti-mouse (P0447; Dako), or rabbit anti-goat (P0449; Dako). The relative density of the immunoreactive bands was analyzed using ImageJ 1.53 software (NIH, Bethesda, USA).

### Functional metabolic assays

Oxygen consumption rate (OCR) and extracellular acidification rate (ECAR) were quantified using a Seahorse XFP analyzer (Seahorse Biosciences) as previously described [21, 22]. Briefly, cells were seeded in XFp miniplates and treated for 24 h with 5 µM CoQ_10_ or vehicle (ethanol). Cells were incubated with Seahorse XFp base medium for 60 min at 37ºC without CO_2_ before loading into the analyzer. To study mitochondrial respiration, OCR was measured under different conditions. Three baseline OCR values were obtained during the first 20 min, after which the different mitochondrial inhibitors were added (oligomycin, 1 μM; carbonyl cyanide-p-trifluoromethoxyphenylhydrazone [FCCP], 0.3 μM; antimycin A and rotenone, 1 μM). Finally, 1 µg/mL Hoechst 33,342 was added, and the total number of cells per well was quantified using a Cytation 5 (Biotek). Respiration parameters were calculated using Wave 2.6 software. A similar protocol was followed for glycolysis, although incubating the cells for 60 min in Seahorse XFp medium base without glucose. Three baseline ECAR measurements were taken for each well within the first 20 min, and glucose (10 mM), oligomycin (1 μM), 2-deoxyglucose (50 μM), and Hoechst 33,342 (1 µg/mL) were subsequently injected for the evaluation of the different parameters. Results were normalized against the total number of cells per well.

#### Boyden chamber/invasion assay

Cell invasion assay was performed using a Boyden chamber with polycarbonate Matrigel-coated membranes, as previously described [15]. Briefly, after treatment, cells were plated into the upper well of the chamber in FBS-free medium, while the lower chamber was filled with FBS-supplemented medium. After 24 h, migrated cells were fixed with 4% PFA; images were acquired using fluorescence microscopy (Nikon TiU) and then analyzed using ImageJ 1.53 software (NIH, Bethesda, USA).

#### Matrix degradation

Cells were treated for 24 h with 5 µM CoQ_10_ or vehicle (ethanol). Then, cells were transferred to coverslips coated with Oregon Green 488-labeled gelatin (G13186; Thermo Fisher), as previously described [23]. After 24 h, coverslips were fixed with 4% PFA and stained with Actin-red (R37112; Thermo Fisher) and DAPI solution. Images were acquired using a Nikon TiU microscope and analyzed using ImageJ 1.53 software (NIH, Bethesda, USA).

#### Quantification of MMPs Activity

EnzChek Gelatinase/Collagenase Assay Kit (E12055; Thermo Fisher) was used to determine MMP 2/9 proteolytic activity. For this purpose, the cells were treated with CoQ_10_ or vehicle (ethanol) for 24 h, after an incubation of 16 h in the dark at 37℃, and a reaction solution with DQ gelatin (E12054; Thermo Fisher) and enzyme (purified collagenase type IV from *Clostridium histolyticum*) was added. Further, determination by fluorimetry (495/515) was also carried out. Results show the ratio of collagenase/gelatinase activity against control cells.

#### Cell tube formation

U251 cells were treated for 24 h with 5 µM CoQ_10_ or vehicle (ethanol; control) in 24-well plates. Conditioned cells medium was transferred to HUVEC cells plated onto GFR Matrigel-precoated 96-well plates. Cells were incubated for 6 h with a conditioned medium, and then the number of cell tubes was quantified using phase-contrast microscopy.

#### Angiogenesis array

For the determination of soluble inflammatory and angiogenic factors, the Human Angiogenesis Array Q2 was used (QAH-ANG-2–1; RayBiotech). The assay was conducted under manufacturer conditions. A full description of the methodology is provided in Supplementary Materials and methods.

#### THP1- HUVEC adhesion

HUVEC cells were incubated with conditioned media of U251 cells treated with vehicle (control) or CoQ_10_. Thp-1 monocytes loaded with Calcein-AM (#C34852; Thermo Fisher) were added to HUVEC monolayers. After a 24-h incubation period, unattached monocytes were removed, and endothelium-adhered monocytes (green) were quantified using fluorescence microscopy (Nikon Ti-U) and analyzed with ImageJ.

#### THP-1 transmigration assay

Thp-1 cells were incubated with conditioned media of U251 cells treated with vehicle (control) or CoQ_10_. Then, a Boyden chamber with untreated polycarbonate membranes was used. The conditioned cells were added to the upper chamber and incubated for 24 h. Then, Thp-1 monocytes that crossed the membrane were fixed, marked with DAPI, quantified using fluorescence microscopy (Nikon TiU) and analyzed with ImageJ.

#### Differentiation of THP-1 to monocytes

Human Thp-1 monocytes were incubated with conditioned media from U251 cells treated with vehicle (ethanol) or CoQ_10_. After 7 days, the cells in suspension were removed, and those that had adhered and differentiated to macrophages were fixed, marked with DAPI, quantified by fluorescence microscopy (Nikon TiU), and analyzed with ImageJ.

#### Statistical analysis

Different statistical analysis was performed using GraphPad Prism 7 software (GraphPad). Data are expressed as mean ± SEM. When comparing two groups, *P* values were calculated using two-tailed Student’s *t-*tests or a one-way ANOVA (Kruskal–Wallis’ test). Differences were considered significant at p < 0.05 (*, *P* < 0.05; **, *P* < 0.01; ***, *P* < 0.001; ****, *P* < 0.0001). Additional methods are described in the Supplementary Materials and Methods.

## Results

### CoQ10 cuts GBM growth

**Fig. 1:**
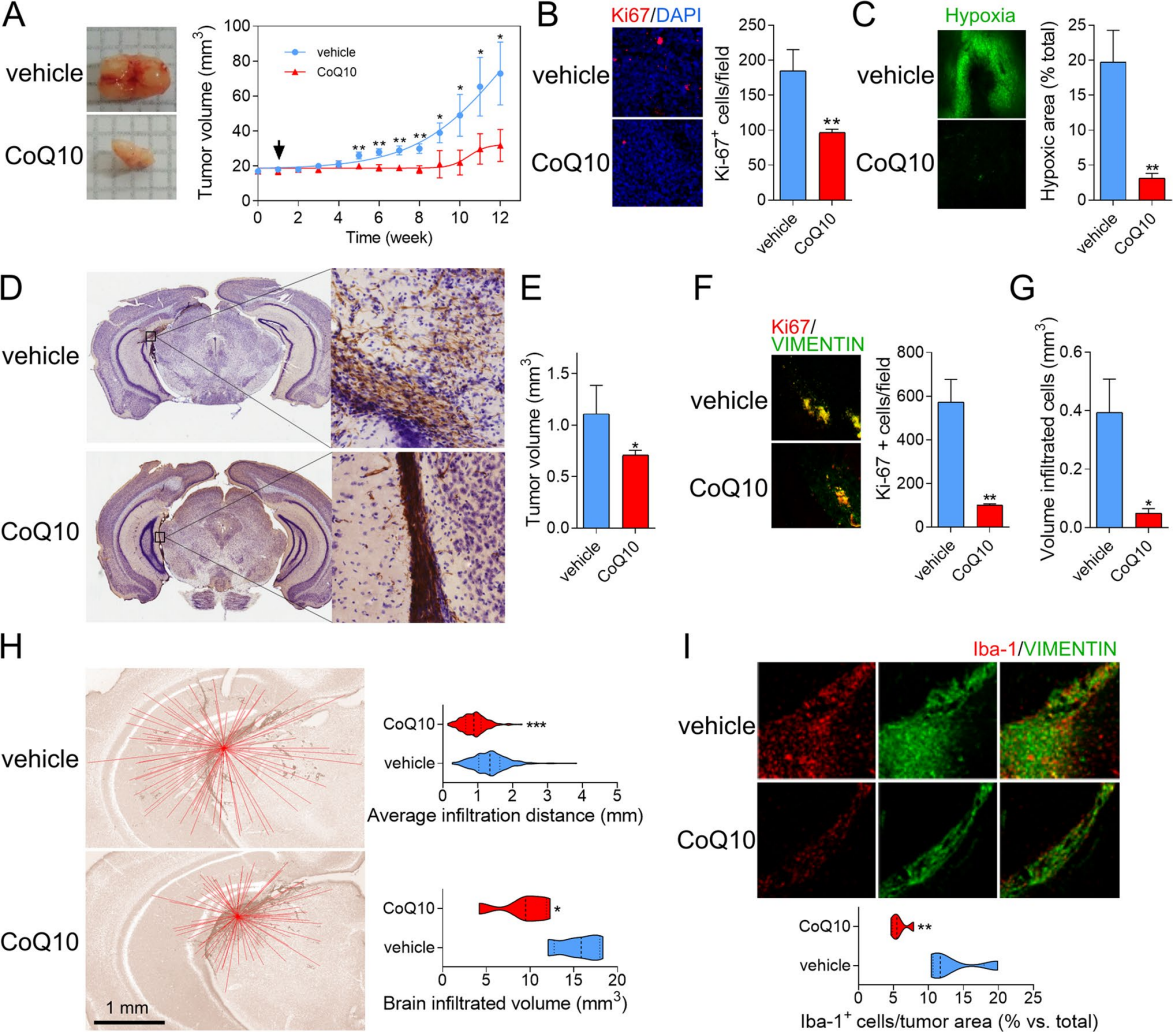
Exogenous CoQ_10_ reduces tumor growth and infiltration in xenografts and orthotopic mouse models of GBM. **A**, U251 cells were implanted subcutaneously into nu/nu mice. CoQ_10_ was injected i.p. every four days starting from the first week (black arrow). Tumor volume was determined weekly with a digital caliper. **B**, Tumors were sectioned and stained with an anti-Ki-67 antibody to quantify proliferation. **C**, Pimonidazole, a hypoxia probe, was injected i.p. prior to euthanasia. Hypoxia was determined by immunodetection of pimonidazole adducts. **D–E**, U251 cells were implanted intracranially into mice using stereotactic procedures. CoQ_10_ was injected i.p. every four days, starting from the first week. Tumor volume was determined using the Cavalieri estimator method with vimentin-immunostained slices. **F**, Slides were stained with an anti-Ki-67 antibody to determine the U251 cells proliferation. **G**, Total volume of U251 infiltrated cells was determined by immunostaining with anti-vimentin. **H**, Average distance, and brain volume occupied by vimentin + infiltrating cells were estimated using ImageJ. **I**, Tumor-infiltrating microglia cells were immunostained with anti-iba-1. *, *P* ≤ 0.05; **, *P* ≤ 0.01; ***, *P* ≤ 0.001

The effect of CoQ_10_ was firstly evaluated in U251 GBM cells xenografted subcutaneously into nude mice, monitoring growth weekly. Administration of CoQ_10_ delayed tumor growth by four weeks vs vehicle (Fig. 1A), which was reflected in a 50% reduction in the proliferation marker Ki-67 (Fig. 1B) and a dramatic reduction of hypoxia—which was almost absent in CoQ_10_-treated tumors (Fig. 1C, Supp. Figure 1). Hypoxic regions in the vehicle mice appeared in the core of the xenografts (Supp. Figure 1A). In contrast, only small hypoxic cell populations were observed in CoQ_10_-treated mice. These results were reproduced in an orthotopic model in which U251 cells were implanted into the striatum of nude mice, and behavior analysis was performed after one month. The open field revealed differences in the exploratory behavior between CoQ_10_ and vehicle groups, indicating affectation due to the tumor (Supp. Figure 2A, B). At this step, animals were euthanized, and tumor cells were stained with anti-human vimentin (Fig. 1D). Volumetric values were obtained using serial cuts, applying the Cavalieri estimator method. This approach showed that CoQ_10_-treated mice had smaller tumors than vehicle-treated ones (Fig. 1E, Supp. Figure 3), which was corroborated by a reduction in Ki-67 levels (Fig. 1F). These results were even more marked when measuring the volume of infiltrated tumor cells (Fig. 1G). Indeed, the most dramatic effect was found in cell invasion. In all instances, infiltrated areas/volumes in vehicle-treated mice were larger than tumor cores, whereas invasion was almost eradicated in CoQ_10_-treated ones (Fig. 1H). Moreover, there was a high density of microglia, Iba-1 positive cells within the Vh-tumors group, which was reduced by 60% by CoQ_10_ (Fig. 1I). Microglia showed a differential inflammatory profile in CoQ_10_-treated mice. A parallel decrease in microglia with a pro-inflammatory phenotype (iNOS +) (Supp. Figure 4A) and an increase in microglia with anti-inflammatory profile (Arg +) (Supp. Figure 4B) were observed in CoQ_10_-treated mice.

### CoQ_10_ remodels the proteome

**Fig. 2:**
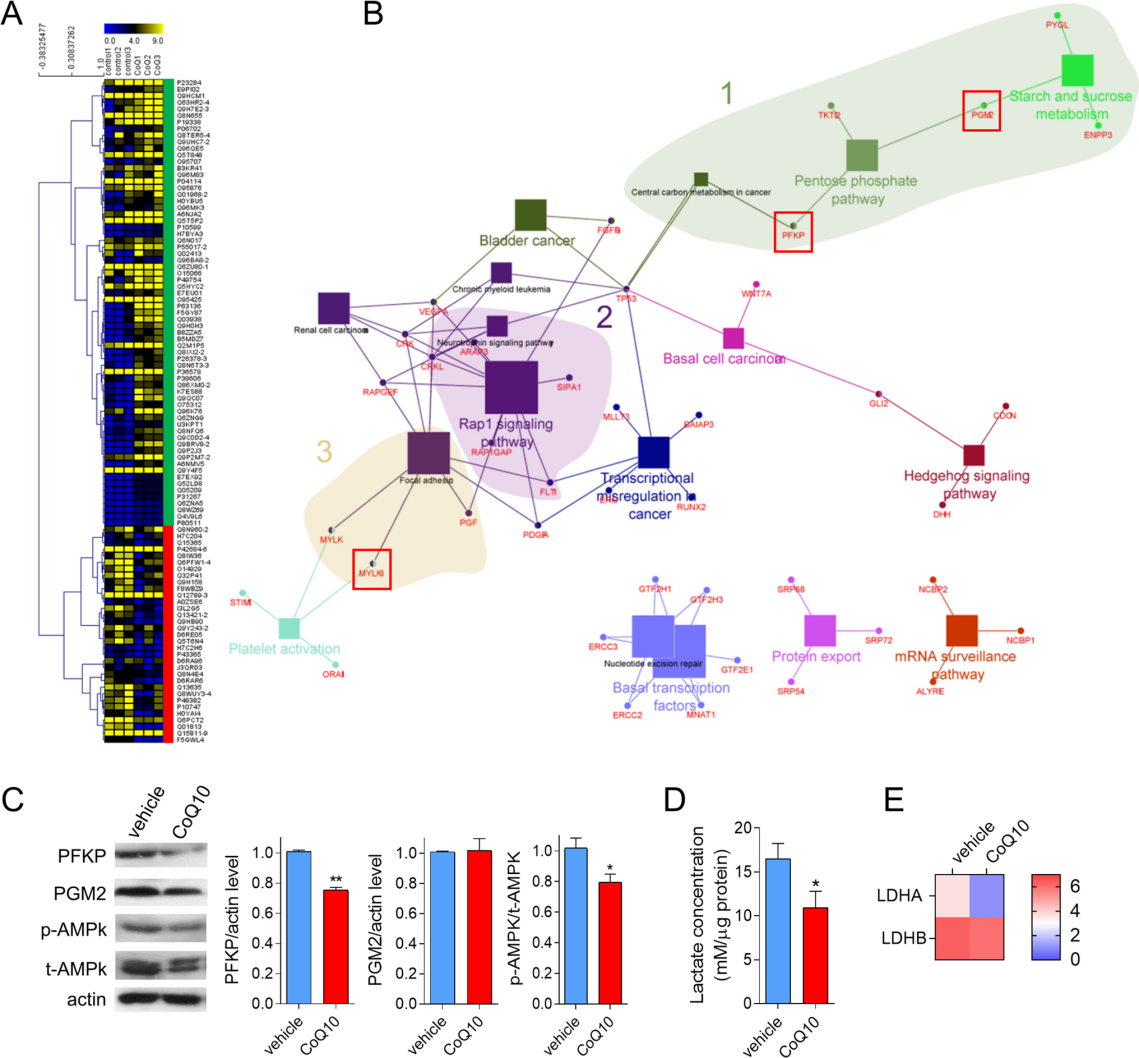
CoQ_10_ remodels human U251 GBM cells proteome in vitro. **A,** U251 GBM cells were incubated with 5 μM CoQ for 24 h. Whole proteome was assessed in a RP-LC–MS/MS using an Easy-nLC II system coupled to a LCQ Fleet ion trap mass spectrometer. **B**, Pathways over-representation was analyzed with the ClueGO v2.3.3 platform. **C**, PFK-P and PGM2 levels were measured by western blot and quantified by densitometry using actin as housekeeping. P-AMPK was normalized vs total level. **D**, Lactate was quantified with a colorimetric L-lactate determination kit (Abcam). **E**, levels of LDHA and LDHB obtained by proteomics from control and CoQ_10_-treated cells for 24 h. *, *P* ≤ 0.05; **, *P* ≤ 0.01

An in vitro proteomics approach was used to deepen the molecular basis of the effect of CoQ_10_ on tumor growth inhibition. A 24-h treatment with 5 μM CoQ_10_ had a notable effect on the whole proteome, significantly altering 150 proteins—with 40 proteins being downregulated and 110 upregulated (Fig. 2A). These protein profiles are framed in key pathways related to tumor growth (i.e., the pentose phosphate pathway and sucrose metabolism), adhesion (i.e., focal adhesion), or angiogenesis (i.e., platelet activation), among others (Fig. 2B). One of the proteins that appeared significantly downregulated was PFKP, a key modulator of glycolysis and gluconeogenesis [24], and thus it was chosen for further quantification with other relevant proteins related to bioenergetics such as PYGL, PGM2, TKTL2, and ENPP3, also altered in proteomics (Supp. Figure 5A, B). The involvement of these proteins in GBM etiology was explored in silico using the Oncomine database (https://www.oncomine.org/resource/login.html). Among the three variants of PFK—platelet, muscle, and liver (PFKP, M and L, respectively)—the first one appeared to be overexpressed in GBM vs white matter (Supp. Figure 5C). Western blot showed that PFKP was downregulated by CoQ_10_ treatment, corroborating the results obtained by proteomics (Fig. 2C). Similarly, levels of the phosphorylated energy sensor AMPK were reduced (Fig. 2C). Also reduced were those of lactate (Fig. 2D), one of the central energy metabolites, and LDHA, but not LDHB (Fig. 2E), reinforcing the idea of an energy remodeling triggered by CoQ_10_.

These results were verified by functional assays using Seahorse XFp. CoQ_10_ treatment significantly reduced basal glycolysis and glycolytic capacity in U251 cells (Supp. Figure 6A), in line with previous results. On the other hand, the application of the MitoStress kit showed that CoQ_10_ had little impact on mitochondrial respiration, generating only a slightly significant increase in basal mitochondrial respiration (Supp. Figure 6B). Finally, proteins related to cell movement and invasion such as C3G and MYLK3 were checked using ICC. Treatment with CoQ_10_ reduced C3G nuclear accumulation and total level (Supp. Figure 7A-B) and MYLK3 accumulation in lamellipodia, perinuclear compartments, and total level (Supp. Figure 7C–D). These results support the idea of cytoskeletal remodeling and cell movement impairment.

### CoQ_10_ reduces cell proliferation and motility

**Fig. 3:**
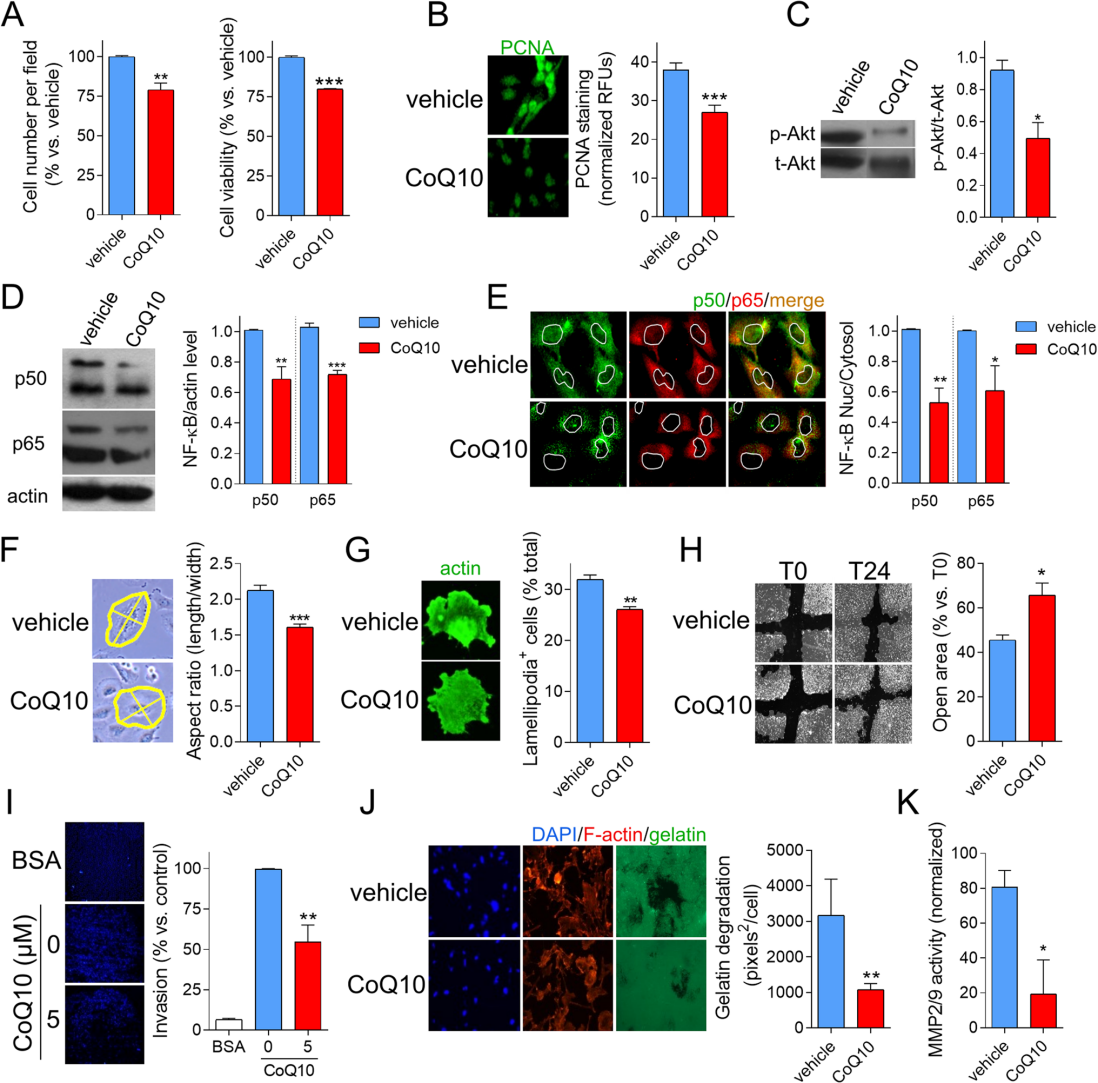
CoQ_10_ impairs cell proliferation, motility, and invasion in vitro. **A**, U251 GBM cells were incubated with 5 μM CoQ_10_ for 24 h. Quantification of the number of attached cells and the percentage of live cells was determined using calcein staining. **B**, Levels of proliferation marker PCNA. **C**, p-/t-Akt was assayed by western blot, quantified by densitometry using ImageJ, and represented as the ratio of p-/t-Akt. **D**, NFκB (p50 and p65 subunits) was determined by western blot. The level of each protein was quantified by densitometry with ImageJ and normalized vs actin. **E**, Representative images of NFκB (p50 and p65 subunits) by immunocytochemistry. The ratio of nuclear/cytosol levels was quantified using ImageJ. **F**, Cell morphology was assessed using phase-contrast microscopy. **G**, Cell motility was evaluated by actin staining and quantification of lamellipodia and **H**, wound healing, calculating the percentage of opened area at 24 h vs T0. **I**, Cells invasion was assessed with a modified Boyden chamber, using Matrigel-coated polycarbonate filters. **J**, Representative images of gelatin degradation patterns and quantification using ImageJ. **K**, MMP2/9 activity quantified using EnzChek Gelatinase/Collagenase Assay Kit. *, *P* ≤ 0.05; **, *P* ≤ 0.01; ***, *P* ≤ 0.001

We have demonstrated in an earlier study that CoQ_10_ reduces the level of HIF-1α in vitro [10]. As this transcription factor is related to cell bioenergetics and bearing in mind the results in vivo, we decided to explore the direct effect of the treatment on tumor expansion in vitro. Cell viability and proliferation were modestly but significantly inhibited by CoQ_10_ treatment (Fig. 3A–B). In addition to HIF-1α, other factors such as Akt or NF-κB, involved in GBM pathophysiology [25–27], are reduced by treatment with the antioxidant. The p-Akt level was reduced by 50% vs control (Fig. 3C), accompanied by a 30% decrease in the total level of NF-κB p50 and p65 subunits (Fig. 3D) and a 50% reduction in their nuclear amount (Fig. 3E). These factors are related to cell proliferation but also motility. Indeed, U251 cells shape tends to be more fibroblast-like in control cells but turns into a more static phenotype upon CoQ_10_ incubation (Fig. 3F), which is mirrored by an inhibition of cell polarization in terms of formation of actin-structures (Fig. 3G). These changes suggested that cell motility could have been affected, and thus cell migration and invasion were evaluated. Classic scratch assay indicated a potent inhibition of cell migration (Fig. 3H) that was also reflected in a robust inhibition of cell invasion through Matrigel (Fig. 3I). This was due to its effect on the actin cytoskeleton but also to the reduction in the activity of MMP2/9—which diminishes the ability to degrade the substrate, necessary for the invasion process (Fig. 3J-K).

### CoQ_10_ inhibits angiogenesis and inflammation

**Fig. 4:**
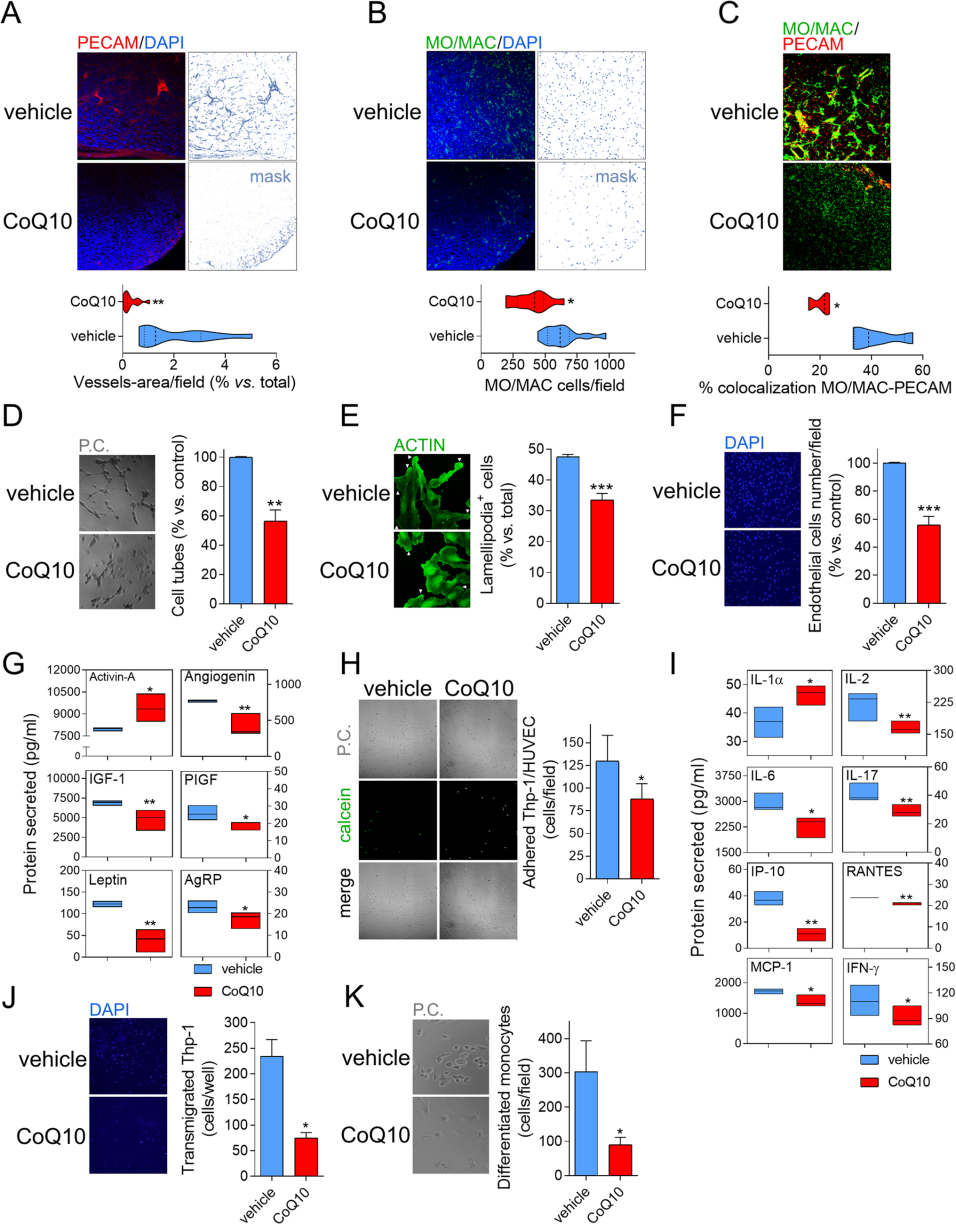
CoQ_10_ reduces neovascularization and inflammation. **A**, Xenografted U251 tumors were sectioned and stained with an anti-PECAM antibody to study the presence of infiltrated vasculature, **B**, and with anti-MO/MAC to determine monocyte and macrophage infiltration. **C**, MO/MAC and PECAM colocalization measured quantified using ImageJ. **D**, Conditioned medium from U251 GBM incubated with 5 μM CoQ for 24 h or control cells, was transferred to HUVEC cells and incubated for 6 h. Representative images and percentage of HUVECs cell tube formation. **E**, Representative images and percentage of HUVECs lamellipodia. **F**, Representative images, and percentage of HUVECs attached cells per field. **G**, Quantification of angiogenesis-related factors measured in conditioned media of U251 cells. **H**, Adhesion of Thp-1 cells to HUVECs growth in U251 conditioned media. Representative images and numbers of cells adhered. **I**, Quantification of inflammation-related factors measured in conditioned media of U251 cells. **J**, Thp-1 cells were incubated in conditioned media of U251 cells (treated with CoQ and control). Representative image and number of cells/ well transmigrated cells using a Boyden chamber with untreated polycarbonate membranes. **K**, Number of monocytes differentiated after 7 days of growing in conditioned media. *, *P* ≤ 0.05; **, *P* ≤ 0.01; ***, *P* ≤ 0.001

CoQ_10_ affected cell growth and motility in vitro, although the effects observed were much weaker than those observed in vivo. As with angiogenesis, microvascular hyperplasia and inflammation influence tumor expansion are controlled by HIF-1α and NF-κB [5–7], both of which are downregulated by CoQ_10_. We thus explored if these processes were affected by the administration of the antioxidant. Tumors from Vh-treated mice were bloody compared to CoQ_10_ ones (Fig. 1A), which was due to high microvessel density, as evaluated by IHC with anti-PECAM (Fig. 4A). As well as the reduction in neovascularization caused by CoQ_10_, we observed an impairment in monocyte and macrophage infiltration (Fig. 4B) and a reduced colocalization of these inflammatory cells and newly formed microvessels (Fig. 4C). To gain more insight into the rationale of these responses to CoQ_10_, in vitro experiments were performed. Conditioned media from U251 cells was added to endothelial cells to perform cell-tube formation assays. Conditioned media from GBM cells incubated with CoQ_10_ inhibited the number of “cell-tubes” (Fig. 4D) due to a direct blockade of endothelial cells polarization, which was measured by quantification of lamellipodia (Fig. 4E), and proliferation, measured by cell counting (Fig. 4F). Thus, we can suggest that angiogenesis in vitro is impaired by conditioned media of U251 cells incubated with CoQ_10_. We next explored the secreted factors contained in the conditioned culture media. Media from CoQ_10_-treated cells had reduced levels of pro-angiogenic factors such as angiogenin, PIGF, leptin, AGRP, and IGF, increasing levels of the antiangiogenic factor activin-A (Fig. 4G). Besides, conditioned media were used to explore some processes related to inflammation in vitro. Conditioned media from cells incubated with CoQ_10_ reduced the adhesion of Thp-1 monocytes to endothelial cells (Fig. 4J), which is related to changes in the inflammatory secretome—evidenced by decreased IL-2, IL-6, IL-17, IP-10, RANTES, MCP-1, and IFN-ɣ, and increased IL-1α (Fig. 4I). Alteration of the inflammatory balance caused a shift to an anti-inflammatory profile, resulting in the inhibition of transmigration of Thp-1 monocytes and the further inhibition of their differentiation to macrophages (Fig. 4J-K).

## Discussion

There has not been any major advance in the treatment of GBM since 2005, when the Stupp protocol was established [1], with an overall median survival of patients between 5 and 15 months [2]. In this study, using in vitro and in vivo models of GBM, we show how CoQ_10_, a lipophilic antioxidant and component of the mitochondrial electron transport chain, exerts pleiotropic effects that control the growth and infiltration of tumor cells. CoQ_10_ can be orally administered, crosses the blood–brain barrier, has very small side effects at therapeutic doses, and is cheap [28, 29].

The uptake of CoQ_10_ in vitro is very swift. Only two hours after its addition to cells, it accumulates into mitochondrial and mitochondrial-associated membranes (MAM), reaching a maximum after 12 h of incubation—at which point it is already incorporated and accumulated in all cellular membranes [30]. CoQ_10_ plays a crucial role in the mitochondrial electron transport chain, thus having a tremendous therapeutic potential. Tumor cells behave as hypoxic and obtain energy mainly by glycolysis [31] producing high leves of O_2_^.−^, a hypoxic signal. O_2_^.−^ stabilizes HIF-1α, promoting the hypoxic response—i.e., glycolysis, tumor cell proliferation, angiogenesis, and evasion of immune response [32]. We have previously reported that CoQ_10_ reduces the mitochondrial O_2_^.−^ and H_2_O_2_ in GBM cells but not in normal astrocytes, as well as a decrease in HIF-1α [10].

In this study, we report how CoQ_10_ remodels the proteome of GBM cells. A decrease in H_2_O_2_ levels and the shift to pro-oxidant conditions promoted by CoQ_10_, as described earlier [10], could be responsible for regulating kinases. In control conditions, Akt and AMPK kinases are activated, which is linked to survival and avoidance of apoptosis [33]. CoQ10 induces mild changes in phosphorylation in many targets simultaneously, leading to a slight inhibition of proliferation and a moderate reduction of motility. Akt and NF-κB are also essential factors in controlling the migration and invasion of GBM, and both are lowered by CoQ_10_ [25, 34, 35]. Indeed, previous evidence indicates that CoQ_10_ has a similar effect on breast cancer cells [36]. Our results show that invasion is also reduced in vivo, with the orthotopic model being the most suitable to explore this process. Brain volume infiltrated by tumor cells was half the size in CoQ_10_-treated mice than in vehicle-treated ones. It is well known that MMP2/9 are critical molecules for regulating the extracellular matrix and invasion of GBM cells [37]. Indeed, both MMPs are targets for the treatment of GBM. Our results indicate that cells treated with CoQ_10_ have reduced motility, but this also impairs their ability to invade by inhibiting MMP2/9 activity.

CoQ_10_ modulates the transcription of hundreds of genes through activation of the transcription factor Nrf-2 [38]. Analysis of the proteome of GBM cells treated for 24 h with CoQ_10_ revealed the up- and downmodulation of more than 150 proteins, related to diverse biological processes—i.e., cytoskeletal reorganization and motility, transcriptional regulation, proliferation, and, more specifically, cell metabolism, including the pentose phosphate pathway (PPP) and sucrose metabolism. Warburg effect or normoxic glycolytic metabolism is characteristic of GBM [32], and tumor cells take advantage of this less energetic variant of glucose metabolism to feed the PPP [32]. Moreover, a high proportion of lactate is secreted, promoting angiogenesis and evasion of the immune response [32]. Our results in this study and previously published [10] show how CoQ_10_ does not significantly improve oxidative phosphorylation in GBM cells, inducing a slight increase in OCR in basal conditions. Instead, it decreases glycolysis and secreted lactate levels. Therefore, it is tempting to propose that the compound partially blocks the Warburg effect. Moreover, CoQ_10_ downregulates PFKP levels—a key enzyme governing glycolysis, which is overactivated in GBM [39] and promotes tumor growth [40].

Although the effects of CoQ_10_ in vitro are moderate, its effect in xenografts and orthotopic models is dramatic due to the regulation of other components of the tumor cell niche, i.e., endothelial and inflammatory cells [32]. The xenograft model allows monitoring the effect of CoQ_10_ rapid and easily. This strategy showed that tumors from CoQ_10_-treated mice were 2.5 times smaller than vehicle-treated ones, which was half-volume in the orthotopic model at the endpoint. This is not comparable to the 25% inhibition of cell proliferation in vitro. This dramatic effect in vivo is due to the inhibition of neovascularization and the blockade of inflammatory cell infiltration. Neovascularization is a crucial target for controlling tumor growth and expansion, and its delay is one of the main goals of GBM treatment. However, modifications of the Stupp protocol implementing bevacizumab (Avastin) had low or no impact on tumor progression [41]. Bevacizumab blocks VEGF-A. Our proteomic results indicate that VEGF level is not altered in GBM cells treated in vitro with CoQ_10_ (not shown). Conversely, CoQ_10_ treatment helps reduce several angiogenic factors such as angiogenin, leptin, and IGF-1, while it increases the antiangiogenic factor activin A [42]. All this point toward HIF-1α and NF-κB [4, 27]. Besides, as described earlier [43], angiogenesis inhibition can also be influenced by the decrease in the level of extracellular lactate mediated by CoQ_10_. Besides, infiltration of inflammatory cells contributes to GBM cells neovascularization and infiltration, degrading the extracellular matrix and secreting interleukins and growth factors [44]. Our results show that CoQ_10_ inhibits monocyte and macrophage infiltration in xenograft models and microglia in orthotopic models. The latter showed a shift in the inflammatory profile of the microglia, being less pro-inflammatory in CoQ_10_-treated mice. However, it is unclear what role this change in phenotype may play in tumour growth but could be key in the smaller tumour volume and less infiltration observed in our study. Patient studies suggested that less inflammatory phenotype can increase survival [45], while an increased presence of pro-inflammatory microglia induce further tumour progression and infiltration [46]. IL-6 produced by GBM cells is essential for inflammatory cell infiltration [44]. This interleukin, together with IL-2 and the monocyte chemoattractant protein 1 (MCP-1), controlled by NF-κB [34], are diminished in the culture media of GBM cells treated with CoQ_10_. Added to the decrease observed in IP-10, RANTES, and IFN-γ, and increase in IL-1α levels, it could explain the inhibitory effect of CoQ_10_ on the infiltration of inflammatory cells in GBM models in vivo, and its repercussion in neovascularization and invasion processes.

Our results provide the first evidence of the efficacy of CoQ_10_ in controlling several hallmarks of GBM, such as growth, invasion, neovascularization, and infiltration of inflammatory cells, as well as the molecular bases affecting these processes. Considering previous results indicating that CoQ_10_ radiosensitizes the tumor with few major side effects in normal astrocytes, we suggest it might be interesting to consider the potential implementation of CoQ_10_ within the Stupp protocol to improve the current treatment of this deadly pathology.

## Supplementary Information

Below is the link to the electronic supplementary material.

Supplementary file1 (DOCX 24 KB)

Supplementary file2 (PDF 4579 KB)

## Data Availability

The datasets used and/or analyzed during the current study are available from the corresponding author upon reasonable request.
