# Supplementary material for: CoQ10 reduces glioblastoma growth and infiltration through proteome remodeling and inhibition of angiogenesis and inflammation
Source: Cell Oncol (Dordr). 2022 Nov 2;46(1):65–77. doi: 10.1007/s13402-022-00734-0 (PMC9947058; doi:10.1007/s13402-022-00734-0)
Supplement: Supplementary file 1 — Supplementary file1 (DOCX 24 KB) [file 13402_2022_734_MOESM1_ESM.docx]

**Supplementary Materials and Methods**

*Mice models*

This study was performed in accordance with Spanish legislation on ‘Protection of Animals Used for Experimental and Other Scientific Purposes’, 86/609/EEC Council on this subject.

*Immunofluorescence and immunohistochemical procedures*

The next primary antibodies were used in immunohistochemistry. Anti-Vimentin (sc-6260; SCBT), anti-Ki-67 (sc-15402; SCBT), anti-Iba-1 (019-19741; Wako), anti-PECAM (sc-8306; SCBT), anti-MO/MAC (sc-59332; SCBT), anti-arginase I (sc-166920; SCBT) and anti-iNOS (sc-727; SCBT).

*Tumor volume and immunohistochemistry estimations*

Volumetric values were obtained using serial cuts, applying Cavalieri Stimator's method [1]. Briefly, once the values for a whole series of section images were obtained, the volumetric approximation is performed following the next equation: Volume= t × a/p × P, where 't' is the section thickness, 'a/p' is the representing area of each point on the point counting grid, and 'P' is the total number of points touching the sections' surface areas. Depending on the volume tumor, up to 9 points were taken per mouse.

To calculate the maximum infiltration volume of cells in orthotopic models, 6 microscopy images of each mouse were selected, and the distance of the 10 most infiltrated cells was calculated for each one using ImageJ. In addition, it was determined whether within this maximum infiltration volume there were infiltrated cells. For this purpose and using masks, the total area occupied by vimentin-positive cells per slice was quantified, and a Cavalieri Stimator’s was applied. To obtain the percentage of Iba-1+ cells, the determination was performed on three sections of each mouse.

*Hypoxia quantification*

To quantify hypoxia levels, intraperitoneal injection of pimonidazole (Hypoxyprobe) at a dose of 60 mg/kg was performed 3h before animal sacrifice. After a sacrifice and tissue processing and cutting, pimonidazole adducts in the hypoxic regions were immunodetected with an anti-MAb1 (1:50) and subsequent labeling with a FITC-conjugated antibody.

*Immunocytochemistry*

The next primary antibodies were used in immunocytochemistry experiments: PCNA (#2586; Cell Signaling), NFkB p50 (sc-1190; SCBT), NFkB p62 (sc-109; SCBT), actin (MA5-11869; Thermo Scientific), C3G (sc-17840; SCBT), MYLK3 (sc-365352; SCBT), p-FIL (#4761; Cell Signaling), t-FIL (sc-17749; SCBT), t-STAT1 (sc-464; SCBT), p-STAT1 (sc-8394; SCBT), t-PRK2 (sc-271526; SCBT) and p-PRK2(#2611; Cell Signaling).

*Western blot*

The following primary antibodies were used for protein quantification by western blot: NFkB p50 (sc-1190; SCBT), NFkB p62 (sc-109; SCBT), p-AKT (sc-135651; SCBT), t-AKT(sc-8312; SCBT), PFKP (sc-514824; SCBT), AMPK (sc-25732; SCBT), PGM2 (sc-376718; SCBT), p-FIL (#4761; Cell Signaling), t-FIL (sc-17749; SCBT), t-STAT1 (sc-464; SCBT), p-STAT1 (sc-8394; SCBT), t-PRK2 (sc-271526; SCBT) and p-PRK2(#2611; Cell Signaling).

*Open field*

The open field assay was performed as previously described [2, 3]. Exploratory activity was assessed for 5 min in the open-field test. Briefly, mice were placed in the middle of the open field maze system (55 × 55 × 25 cm high – white color, wooden) and observed for 5 min. Horizontal crossings of squares (segments) and total distance were recorded using a video-camera system and then analyzed.

*Viability*

Viability assay was performed as previously described [4]. U251 cells were seeded in 96-well plates and treated 24 h with 5 µM CoQ_10_ or vehicle (ethanol; control). After that, cells were incubated with (#46067; Sigma-Aldrich) and 1 μM Calcein-AM (#C34852; Thermo Fisher). Viable (green), necrotic cells (red), and cell number were determined by fluorescence microscopy with a Nikon TiU microscope (Nikon, Tokyo, Japan) using a 20× objective and then quantified using Image J 1.53 software (NIH, Bethesda, USA) (n = 3).

*Angiogenesis array*

U251 cells were treated for 24 h with 5µM CoQ_10_ or vehicle (ethanol) and lysed with 2X Cell Lysis buffer. Briefly, lysed cells were blocked for 60 min. The array must be previously equilibrated at room temperature for 30 minutes. A negative control (culture medium supplemented with FBS) must be included among the samples. Samples and standards were incubated overnight with sample diluent, washed twice and incubated with detection antibody solution for two hours. After which, it is washed 5 times in a washing solution to remove debris. Then, Cy3 equivalent dye-conjugated streptavidin was added to each well and incubated in the dark for one hour. Finally, the incubation device was disassembled, taking care not to touch the slide, and a new series of washes were performed. After carefully drying the slide, it was analyzed using a Genepix array reader. The levels of each molecule were obtained in pg/mL, interpolating on the different standard curves.

*Cell size*

U251 cells were treated 24 h with 5 µM CoQ_10_ or vehicle (ethanol; control). After cell treatment, bright field images were acquired in fluorescence microscopy (Nikon TiU) using a 20x objective. The aspect ratio was measured by length and width were quantified using ImageJ 1.53 software (NIH, Bethesda, USA) in 100 independent cells per condition.

*Migration assays*

As previously described, wound healing and cytoskeleton reorganization assays were performed [4]. U251 cells were plated in 24-well, cultured to confluence, and then incubated in serum starvation conditions for 24h in medium with 5 µM CoQ_10_ or vehicle (ethanol; control). A cross-scratch was done in the cell monolayer with a 200 µL pipet tip, then the medium was replaced by fresh medium. Percentage of wound closure was calculated using Image J 1.53 software (NIH, Bethesda, USA), measuring the open rea free of migrated cells immediately after the scratch and at 24h (n = 3).

**References**

1 B.Z. Altunkaynak, E. Altunkaynak, D. Unal and B. Unal, Eurasian J Med 41, 99-101 (2009)

2 V. Torres-Lista, C. Parrado-Fernández, I. Alvarez-Montón, J. Frontiñán-Rubio, M. Durán-Prado, J.R. Peinado, B. Johansson, F.J. Alcaín and L. Giménez-Llort, Behavioural brain research 271, 140-146 (2014) doi: 10.1016/j.bbr.2014.04.055

3 L. Giménez-Llort, Y. García, K. Buccieri, S. Revilla, C. Suñol, R. Cristofol and C. Sanfeliu, International journal of Alzheimer's disease 2010, 1-17 (2010) doi: 10.4061/2010/128354

4 M. Duran-Prado, J. Frontinan, R. Santiago-Mora, J.R. Peinado, C. Parrado-Fernandez, M.V. Gomez-Almagro, M. Moreno, J.A. Lopez-Dominguez, J.M. Villalba and F.J. Alcain, PloS one 9, e109223 (2014) doi: 10.1371/journal.pone.0109223
