# Supplementary material for: CoQ10 reduces glioblastoma growth and infiltration through proteome remodeling and inhibition of angiogenesis and inflammation
Source: Cell Oncol (Dordr). 2022 Nov 2;46(1):65–77. doi: 10.1007/s13402-022-00734-0 (PMC9947058; doi:10.1007/s13402-022-00734-0)
Supplement: Supplementary file 2 — Supplementary file2 (PDF 4579 KB) [file 13402_2022_734_MOESM2_ESM.pdf]

Supplementary Figures

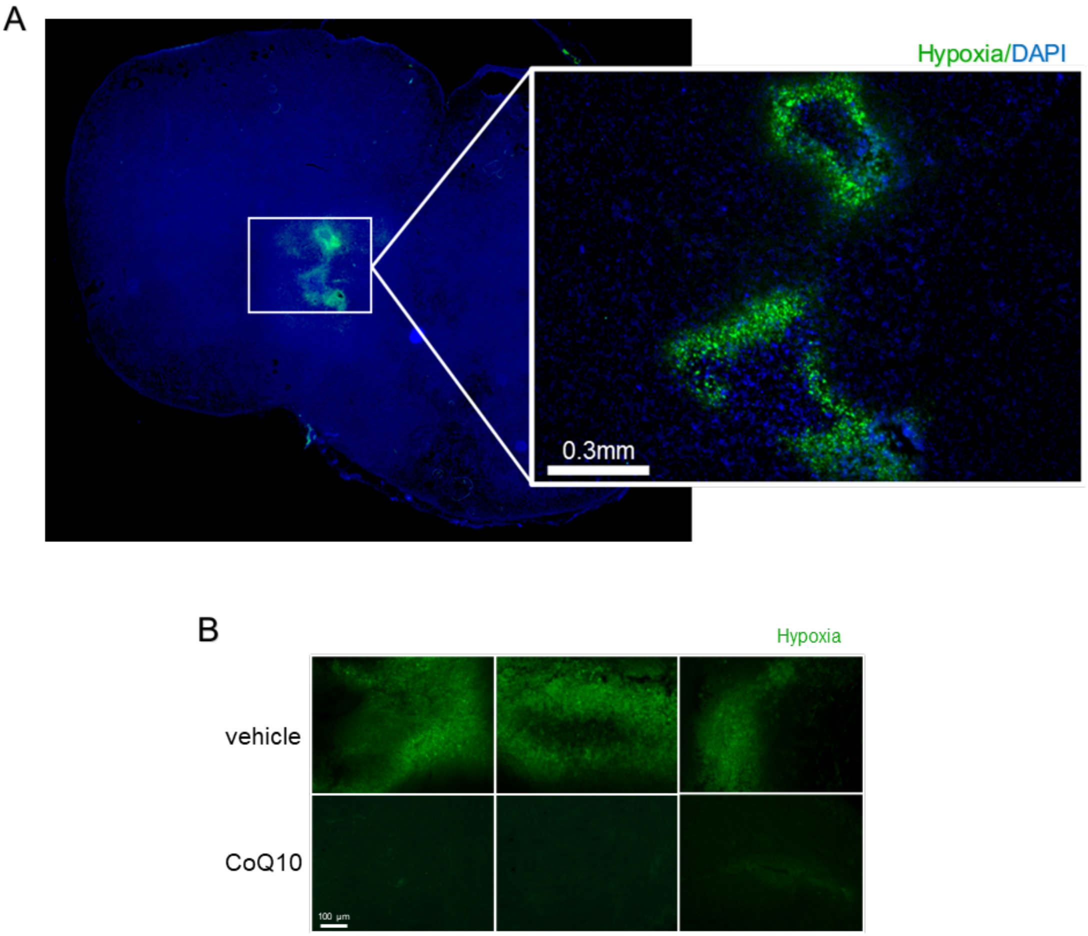

Supp. Fig. 1. CoQ10 reduces tumor hypoxia in xenografts. A, Pimonidazole was injected via i.p., preceding animal`s euthanasia. Hypoxia was determined by immunodetection of pimonidazole adducts. Scan of a xenograft with DAPI (blue, nuclear labelling) and hypoxic regions (green) and B, fluorescence microscopy image showing the hypoxic areas in detail.

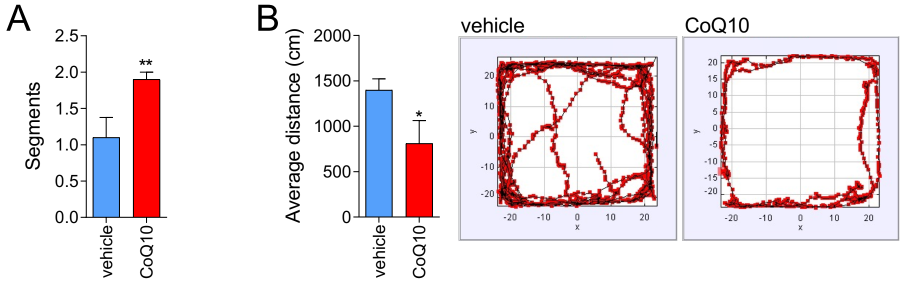

Supp. Fig. 2. Delay of tumor growth in orthotopic models of GBM is reflected in improved skills. A, The number of segments was determined using a standard static rod test. B, Average distance covered and representative movements distribution of open field test.

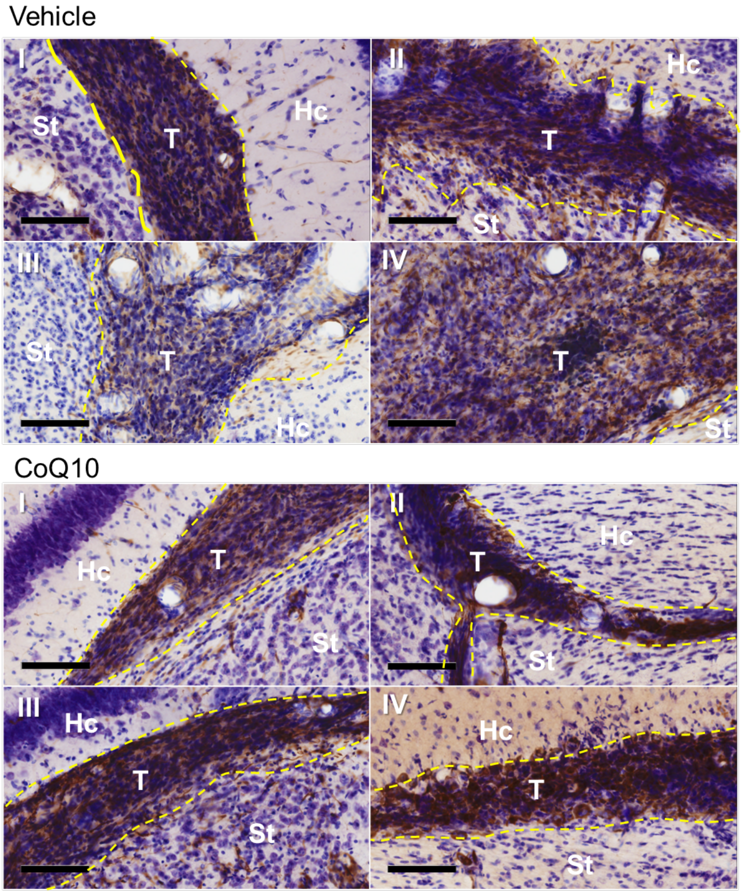

Supp. Fig. 3. Tumors (yellow lines - T) were in the upper striatum (St). Tumor cells invaded part of the striatum and hippocampus (Hc). Scale bar = 100 µm.

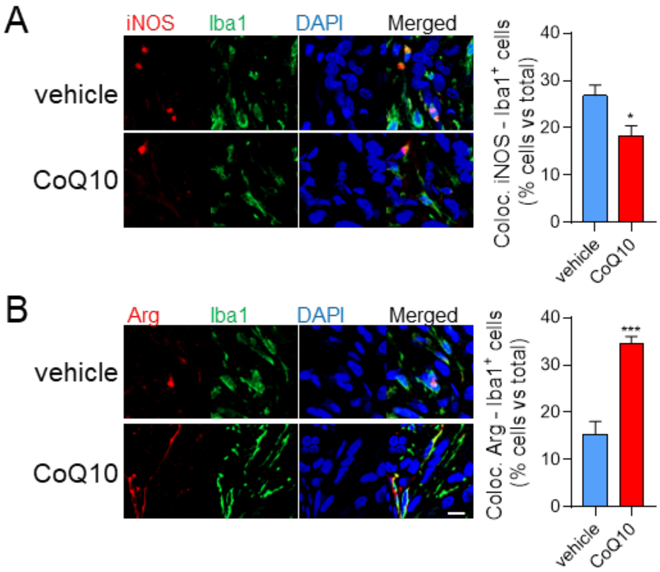

Supp. Fig 4. CoQ_{10} induces a phenotypic microglial inflammatory shift in an orthotopic model of GBM. A, U251 cells were implanted intracranially into mice using stereotactic procedures. Percentage of iNOS + cells was determined by immunostaining with anti-iNOS antibody. B, Percentage of arginase I + cells was determinaed by immunostaining with anti-arginase I antibody.

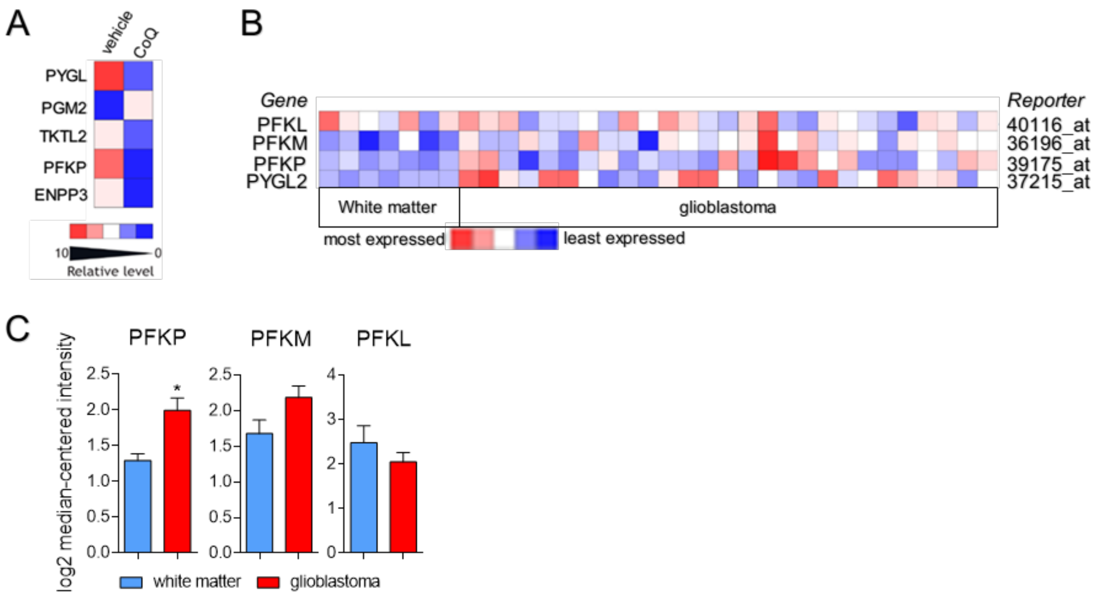

Supp. Fig. 5. In silico analysis of metabolic targets in human GBM series. A, levels of PYGL, PGM2, TKTL2, PFKP and ENPP3 obtained by proteomics from control and CoQ_{10}-treated cells for 24h. B, Oncomine analysis of PFKL, PFKM, PFK2 and PYGL2 in human brain series including glioblastoma and non-tumor white matter. C, Quantification of the P, M and L isoforms of PFK in human brain series including glioblastoma and non-tumor white matter (https://www.oncomine.org/resource). *, $p<0.05$.

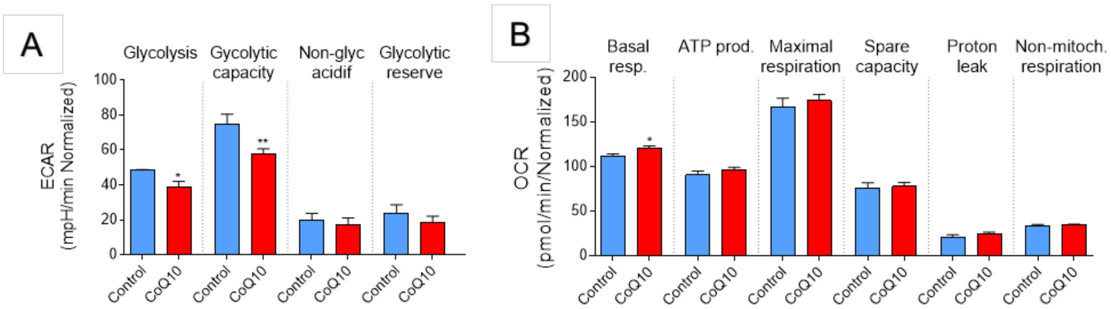

Supp. Fig. 6. U251 GBM cells were incubated with 5 µM CoQ_{10} for 24h. A, Quantification of glycolysis, glycolytic capacity, non-glycolytic acidification and glycolytic reserve by Seahorse XFp. B, Quantification of basal respiration, ATP production, maximal respiration, spare capacity, proton leak and non-mitochondrial respiration by Seahorse XFp.

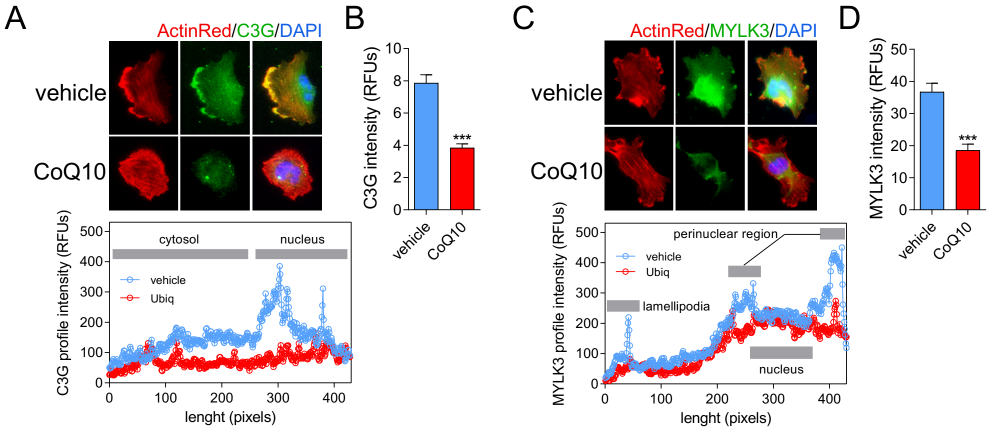

Supp. Fig. 7. Validation of proteomics targets by ICC: U251 GBM cells were incubated with 5 μM CoQ_{10} for 24h. A, Representative images of immunocytochemistry of anti-C3G antibody, actin red and DAPI, and profile intensity analyzed with ImageJ. B, Representative images of immunocytochemistry of anti-MYLK3 antibody, actin red and DAPI, and profile intensity analyzed with ImageJ. ***, $P \leq 0.001$.
